# SARS-CoV-2 infection and the antiviral innate immune response

**DOI:** 10.1093/jmcb/mjaa071

**Published:** 2020-12-30

**Authors:** Hui Yang, Yingying Lyu, Fajian Hou

**Affiliations:** 1 Department of Neurosurgery, Huashan Hospital, Institute for Translational Brain Research, MOE Frontiers Center for Brain Science, Shanghai Medical College, Fudan University, Shanghai 200032, China; 2 State Key Laboratory of Molecular Biology, Shanghai Institute of Biochemistry and Cell Biology, Center for Excellence in Molecular Cell Science, Chinese Academy of Sciences; University of Chinese Academy of Sciences, Shanghai 200031, China; 3 School of Life Science, Hangzhou Institute for Advanced Study, University of Chinese Academy of Sciences, Hangzhou 310024, China; 4 Shanghai Key Laboratory of Brain Function Restoration and Neural Regeneration, Huashan Hospital, Shanghai Medical College, Fudan University, Shanghai 200032, China

The severe acute respiratory syndrome coronavirus 2 (SARS-CoV-2) outbreak began in December 2019, causing the illness known as the novel coronavirus disease 2019 (COVID-19). The virus spread rapidly worldwide to become a global public health emergency. As of November 15, 2020, more than 53 million confirmed cases and over 1 million deaths worldwide have been reported (World Health Organization, 2020). The SARS-CoV-2 genome was sequenced and studies are ongoing to further understand the epidemiology, clinical manifestations, etiological structure, cellular receptor angiotensin II converting enzyme (ACE2), and intracellular replication process of the virus. Currently, thousands of clinical trials related to SARS-CoV-2 are underway (https://clinicaltrials.gov/). However, no vaccines or drugs have yet been approved, until very recently, for direct treatment or prevention of COVID-19 and only supportive treatment has been applied clinically. This review will discuss the possible mechanism of the innate immune response to SARS-CoV-2 infection and provide insight into the development of related therapeutics.

## SARS-CoV-2 genome and viral proteins

SARS-CoV-2 belongs to the *Coronaviridae* family, which is a group of enveloped, non-segmented, and positive-stranded RNA viruses with genomes of ∼30 kilobases (Bar-On et al., 2020). The *Coronaviridae* family includes many viruses that infect wild animals, six of which also infect humans. Infection by some of these coronaviruses causes only mild respiratory symptoms in humans. However, SARS-CoV-2, the Middle East respiratory syndrome CoV (MERS-CoV), and SARS-CoV cause severe respiratory diseases. These three viruses belong to the beta-CoV genera, which have different epidemiology despite sharing high genomic and structural similarities (Cui et al., 2019; Wen et al., 2020; Ye et al., 2020).

**Figure 1:**
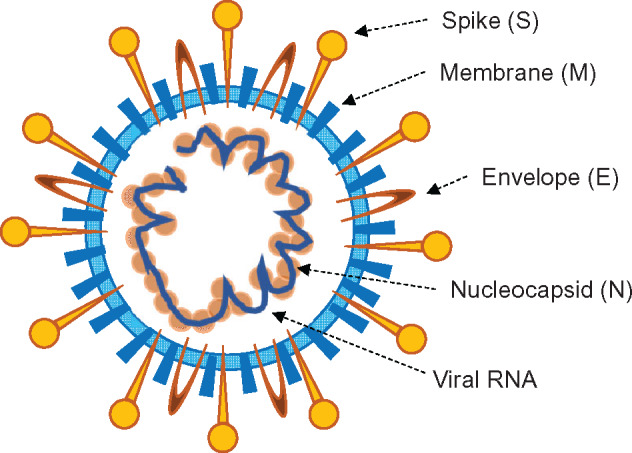
The coronavirus particle. Their envelope contains four structural components, including spike (S), envelope (E), membrane (M), and nucleocapsid (N) proteins inside the virion that covers the viral RNA.

Coronaviruses are spherical in shape with a diameter of 80–120 nm. The most prominent feature of coronaviruses is the club-like projections on the virus surface termed ‘spikes’ (Figure 1). The virion contains four structural components, including spike (S), envelope (E), membrane (M), and nucleocapsid (N) proteins, the last of which binds to the RNA inside the virion. These proteins are encoded by the 3ʹ end of the genome. The coronavirus genome also encodes two polyproteins, pp1a and pp1b, which contain the non-structure proteins required for genome replication; located at the 5ʹ end, they comprise about two thirds of the genome (Snijder et al., 2003; Fehr and Perlman, 2015). At the C-terminus of the well-studied S protein is a receptor-binding domain (RBD) that enables viral binding to the host cell membrane. The main receptor for SARS-CoV and SARS-CoV-2 on the host cell membrane is ACE2, which has a high binding affinity with S protein. This specificity creates epitopes that are recognized by T and B cells to generate neutralizing antibodies (Walls et al., 2020). Following RBD binding to ACE2, an irreversible conformational change of S protein occurs, inducing S protein cleavage into S1 and S2. This process facilitates the fusion of the virus with the target membrane, thus allowing viral RNA to enter the cytoplasm of the target cell (Walls et al., 2020). The amino acid sequence of the SARS-CoV-2 RBD shares a 74% homology to that of SARS-CoV, suggesting similar cell entry mechanisms for the two viruses. These data indicate that ACE2 may be a potential target for COVID-19 therapy.

## Innate immune response

**Figure 2:**
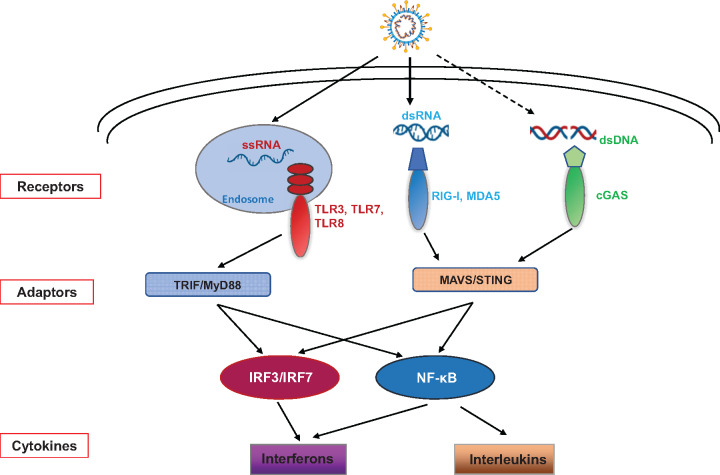
The innate immune signaling pathways sensing SARS-CoV-2. SARS-CoV-2 could be detected by RLR and TLR pathways, and cGAS pathway might also be involved, resulting in the activation of innate immune responses.

The first line of defense against viral infection is innate immune signaling. Pattern-recognition receptors (PRRs) are located on the plasma membranes, endosomal membranes, and in the cytosol to recognize viral components or replication intermediates known as pathogen-associated molecular patterns (PAMPs). PRRs respond to viral PAMPs including lipoproteins glycoproteins and nucleic acids to initiate an antiviral response (Figure 2). Immune cells express several classes of PRRs that detect viral components: toll-like receptors (TLRs), nucleotide oligomerization domain (NOD)-like receptors (NLRs), retinoic acid-inducible gene I (RIG-I)-like receptors (RLRs), and cyclic guanosine monophosphate–adenosine monophosphate (cyclic GMP–AMP, cGAMP) synthase (cGAS) (Tan et al., 2018).

The RLRs are cytoplasmic sensors that detect viral RNAs, including RIG-I, melanoma differentiation-associated gene 5 (MDA5), and laboratory of genetics and physiology 2 (LGP2) (Tan et al., 2018). RIG-I recognizes viral RNA containing a di- or triphosphate at the 5ʹ end, while MDA5 has an affinity for longer dsRNA ligands (Tan et al., 2018). RLR signaling activates several transcription factors, including IRF3, IRF7, and NF-κB. The IRF3 and IRF7 transcription factors initiate type I interferon (IFN-I, including IFNα and IFNβ) expression, which is important for the antiviral response. NF-κB induces the expression of proinflammatory cytokines. Mouse hepatitis virus (MHV), a coronavirus, is detected by RIG-I in brain macrophages and microglial cells (Roth-Cross et al., 2008). MHV, SARS-CoV, and SARS-CoV-2 share similar PAMPs, making them likely to be detected by RLRs. SARS-CoV infection induces NF-κB targeted genes, like IL-6 and IL-8, early in the infection, while IFN-I induction by IRF3 and IRF7 is delayed until 48 h after infection (Yoshikawa et al., 2010).

The TLRs belong to a conserved family of membrane-bound innate immune sensors with the human innate immune system encoding 10 members. Four of the 10 TLRs, TLR3, TLR7, TLR8, and TLR9, recognize RNA viruses, including both double-stranded RNA (dsRNA) and single-stranded RNAs (ssRNA) (Tan et al., 2018). The endosome-localized TLR3 and TLR7/TLR8 recognize RNA viruses and activate IRF3/IRF7 through MyD88/TRIF signaling to induce IFN-I production (Tan et al., 2018). Several studies show that in animal models, the activation of TLR3 facilitates a protective effect against SARS-CoV and MERS (Birra et al., 2020). Moreover, knocking out the adaptor molecule MyD88 in mice is also protective in the MA15-SARS-CoV model (Birra et al., 2020). TLR4, however, resides on the cell surface where it recognizes viral glycoproteins and is a putative respiratory virus co-receptor (Marchant et al., 2010). Additionally, TLR4 is protective in the MHV-1 SARS respiratory model and over-activation of TLR4 by phospholipids is linked to lung damage and increased levels of IL-6 in COVID-19 patients (Birra et al., 2020). Consequently, blocking IL-6 with humanized monoclonal antibodies may be a good treatment for patients with severe symptoms (van Kraaij et al., 2020). A more recent study reported that TLR7 loss-of-function variants were found in four young male patients with severe COVID-19 (Birra et al., 2020).

The cytosolic DNA sensing pathway is initiated by cGAS when bound by dsDNA from viruses or other pathogens. Activation of cGAS produces the secondary messenger, cGAMP, which in turn activates STING to induce IFN-I and proinflammatory cytokines (Ablasser and Chen, 2019). The activation of the DNA sensing pathway is also mediated by some RNA viruses, such as Dengue virus (DENV) and West Nile virus (WNV) (Aguirre et al., 2017). The serine residue 366 of STING is important to activation of the TBK1‒IRF3 signal axis to induce IFN-I production. This serine is located at residue 358 in bats, resulting in a STING allele that is less effective at producing IFN-I (Xie et al., 2018). Consequently, it has been reported that bats have a higher capacity to coexist with coronavirus (Watanabe et al., 2010). However, this correlation does not indicate that bats benefit from a lower innate immune response than humans, since SARS-CoV-2, as an RNA virus, is more likely to be sensed by the RLR‒MAVS pathway than the cGAS‒STING pathway. STING is primarily expressed in lung endothelial cells, spleen, and epithelial cells, which are important for the pathogenicity of SARS-CoV-2 infection. In the late stages of COVID-19, damaged DNA released into the cytosol might activate the cGAS‒STING signaling pathway, resulting in a severe cytokine storm in some patients. Further studies are needed to explore the connection between the activation of the cGAS‒STING pathway and coronavirus infection.

## Development of therapeutics against SARS-CoV-2

Currently, there are no drugs specific for the treatment of SARS-CoV-2. Several classes of drugs including antimalarial agents, IFNs, nucleic acid synthesis inhibitors, and sterol metabolism inhibitors are effective both *in vitro* and *in vivo* against SARS-CoV and MERS-CoV infection (Maurya et al., 2020). Based on previous studies investigating the molecular mechanism of SARS pathogenesis, repurposing current anti-viral agents for COVID-19 treatment reveals that IFNs, antimalarial agents, and nucleoside analogues could be potential strategies against SARS-CoV-2 infection (Table 1).

**Table 1:** Therapeutic strategies against SARS-CoV-2.

Therapeutic agent	Feature	Mechanism	Efficacy
IFN-I (Sallard et al., 2020)	Early cytokines produced upon viral infection; many immunomodulatory properties	Induce interferon-stimulated genes through the IFNAR receptor to interfere with viral replication and activate the adaptive immunity	SARS-CoV-2 is sensitive to IFN-I treatment; recommended in the COVID-19 treatment guideline in China
Chloroquine (Devaux et al., 2020)	Immunomodulatory activity; long half-life (40‒60 days)	Inhibit endosome-mediated viral entry and interfere with the post-translational modification of viral proteins	Inhibit SARS-CoV-2 in cells; discrepancies in clinical outcomes
Remdesivir (Eastman et al., 2020; Saha et al., 2020)	Inhibition of several RNA viruses depending on the RdRp	Inhibit the viral RdRp as an adenosine nucleoside triphosphate analogue	Antiviral efficacy against SARS-CoV-2 in cells; discrepancies in clinical outcomes

### Type I interferon

IFN-I is critical to viral clearance, including the clearance of coronavirus. Many viruses, including SARS-CoV-2, however, have evolved a mechanism to inhibit IFN production and the antiviral effects in the host cell (Sallard et al., 2020). Accordingly, patients in the early stages of SARS-CoV-2 infection showed a limited IFN-I response, whereas IL-6 and other chemokines were elevated (Blanco-Melo et al., 2020). More recently, a study found that the IFN-I responses are abrogated in peripheral blood from severe COVID-19 patients, a contrast to the high IL-6 and TNFα levels observed (Hadjadj et al., 2020). However, several lines of evidence have shown elevated IFN-I levels associated with a strong NF-κB-driven inflammatory response in patients with severe COVID-19 (Lee et al., 2020). While conflicting results on IFN-I in COVID-19 patients have been reported, the use of IFNβ to treat early-stage COVID-19 has had a positive response from several clinical trials (NCT04276688) and more studies are in progress that include the use of IFNα and IFNγ (NCT04343976; Wang et al., 2020a). However, high IFN-I levels may increase cytokines when cytokine storms are a hurdle in treating severe cases of COVID-19. Therefore, the timing and dose of IFN-I treatments must be carefully studied. In addition, the identification of IFN-I-related biomarkers may be valuable in optimizing clinical treatments of severe COVID-19 patients.

#### Chloroquine and remdesivir

Chloroquine and its derivatives are widely used as immunomodulators to treat influenza, seasonal CoVs, and SARS, presumably through the inhibition of innate immune molecules, TLR7 and TLR9 (Lamphier et al., 2014; Devaux et al., 2020).

A previous study showed that chloroquine has an antiviral effect against SARS-CoV-2 *in vitro* (Wang et al., 2020a). This result led to the immediate application of chloroquine to treat COVID-19 patients. One of the first clinical studies providing evidence for the efficacy and safety of chloroquine to treat COVID-19 was conducted in China with a small group of patients (Huang et al., 2020b). All the 10 patients treated with chloroquine in this study were discharged after 14 days of treatment, compared with only six patients (50%) from the Lopinavir/Ritonavir group. This promising result was soon confirmed by a multicenter, prospective, observational study with a larger cohort size (Huang et al., 2020a). However, the use of chloroquine was challenged by several other studies, including the largest retrospective study, which was conducted in the USA. They reported that hydroxychloroquine alone, or in combination with azithromycin, did not show benefit in either viral clearance or clinical outcome (Cavalcanti et al., 2020; Hoffmann et al., 2020; Maisonnasse et al., 2020).

Nucleoside analogues are widely used drugs to treat viral infections. Remdesivir is an anti-viral drug designed to treat Ebola, but may also be effective against MERS and SARS (Sheahan et al., 2020). Soon after the COVID-19 outbreak, remdesivir was also successfully tested for its ability to inhibit SARS-CoV-2 infection *in vitro* (Wang et al., 2020a). Treatment of a small group of patients with remdesivir significantly improved clinical outcomes and reduced mortality (Grein et al., 2020). Due to the seriousness of the SARS-CoV-2 pandemic, remdesivir was quickly granted an emergency use authorization for patients with severe COVID-19 by the US Food and Drug Administration (FDA). But a randomized, double-blinded clinical trial of remdesivir as a COVID-19 therapeutic did not demonstrate efficacy (Wang et al., 2020b). However, a larger clinical trial, the Adaptive COVID-19 Treatment Trial (ACTT-1), randomized the treatment of 1063 patients and reported that remdesivir treatment improved patient recovery (Beigel et al., 2020). A more recent study reported that remdesivir has modest clinical benefits compared with standard care (Spinner et al., 2020). With more clinical trials ongoing, remdesivir may yet be shown as an effective therapeutic for COVID-19.

## Future directions

**Figure 3:**
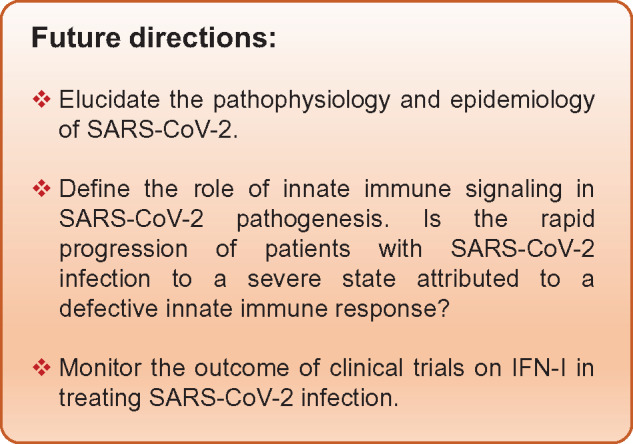
Future directions.

The biology, pathophysiology, and epidemiology of SARS-CoV-2 need further investigation. It is still unclear how patients with SARS-CoV-2 infection progress to a severe state in such a short period of time (Figure 3). Though over-activation of the host immune system may cause serious damages to organs and tissues (and lead to patient death), innate immunity may also play an important role in SARS-CoV-2 infection. Studies of the molecular and cellular signaling mechanisms underlying SARS-CoV-2 infection will illuminate putative COVID-19 treatment in the future.

*[H.Y. was supported by the Program for Professors of Special Appointment (Eastern Scholar) at the Shanghai Institutions of Higher Learning (SSF151005). This work was supported by the National Natural Science Foundation of China (82073166 to H.Y.), the National Key Research and Development Program of China (2020YFA0804200), the Shanghai Municipal Science and Technology Major Project (2018SHZDZX01), and the Zhangjiang Lab.]*
